# A randomized trial of low-dose thrombolysis, ultrasound-assisted thrombolysis, or heparin for intermediate-high risk pulmonary embolism—the STRATIFY trial: design and statistical analysis plan

**DOI:** 10.1186/s13063-024-08688-4

**Published:** 2024-12-28

**Authors:** Jesper Kjærgaard, Jørn Carlsen, Emilie Sonne-Holm, Sebastian Wiberg, Lene Holmvang, Jens Flensted Lassen, Rikke Sørensen, Dan Høfsten, Peter Sommer Ulriksen, Samir Jawad, Pernille Palm, Jens Jakob Thune, Christian Hassager, Ole P. Kristiansen, Kristian Eskesen, Søren Fanø, Lia E. Bang

**Affiliations:** 1 Department of Cardiology, The Heart Centre, Copenhagen University Hospital Rigshospitalet, Copenhagen, Denmark; 2 Department of Cardiothoracic Anesthesiology, Copenhagen University Hospital Rigshospitalet, Copenhagen, Danmark; 3 Department of Cardiology, Odense University Hospital, Odense, Denmark; 4 Departpent of Radiology, Copenhagen University Hospital Rigshospitalet, Copenhagen, Danmark; 5 Department of Cardiology, Copenhagen University Hospital Bispebjerg and Frederiksberg, Copenhagen, Danmark; 6 Department of Cardiology, Copenhagen University Hospital Gentofte, Copenhagen, Danmark; 7 Department of Cardiology, Copenhagen University Hospital Herlev, Copenhagen, Danmark

**Keywords:** Pulmonary embolism, Intermediate-high risk, Low-dose thrombolysis, Ultrasound-assisted thrombolysis, Clot burden

## Abstract

**Background:**

Intermediate-high risk pulmonary embolism (PE) carries a significant risk of hemodynamic deterioration or death. Treatment should balance efficacy in reducing clot burden with the risk of complications, particularly bleeding. Previous studies on high-dose, short-term thrombolysis with alteplase (rtPA) showed a reduced risk of hemodynamic deterioration but no change in mortality and increased bleeding complications. Catheter-based techniques, including ultrasound-assisted thrombolysis (USAT), and low-dose thrombolysis may offer reasonable efficacy with lower risk. However, studies comparing these methods have been few. This trial aims to address this gap by randomizing patients to three treatment modalities.

**Methods:**

Multicenter, randomized trial with 1:1:1 allocation of 210 patients with acute intermediate-high risk PE, excluding those with absolute contraindications to thrombolysis. Patients are eligible for inclusion if they are > 18 years of age, have had symptoms < 14 days, and are able to give informed consent. Patients are allocated 1:1:1 into three treatment strategies: (1) unfractionated heparin (UFH)/low molecular weight heparin (LMWH), (2) UFH/LMWH + 20 mg rtPA/6 h intravenously (IV), or (3) UFH + 20 mg rtPA/6 h via USAT. Co-primary outcomes include reduction in clot burden as assessed by refined Miller score from pre-treatment to follow-up (48–96 h) computed tomography pulmonary angiogram (CTPA) comparing low-dose rtPA (± USAT) groups to UFH/LMWH group (*p* < 0.01, *N* = 210) and reduction in refined Miller score on follow-up CT angiography comparing low-dose rtPA by USAT to intravenous rtPA, *p* < 0.04, *N* = 140). Secondary outcomes comprise bleeding complications, duration of index admission, FiO_2_, blood pressure, respiratory and heart rate at the time of follow-up CT angiography, mortality in the three groups, incidence of tricuspid regurgitation pressure gradient < 40 mmHg at 3 months follow-up echocardiography, 6-min walk test at 3 months comparing the three groups, and health-related quality of life at 3 months follow-up comparing the three groups.

**Discussion:**

We hypothesize that in patients with intermediate-high risk PE (1) administration of 20 mg rtPA leads to a greater reduction in clot burden compared to heparins and (2) administration of 20 mg rtPA via USAT results in a greater reduction in clot burden compared to 20 mg rtPA intravenous.

**Trial registration:**

ClinicalTrials.gov NCT04088292. Registered in September 2019 (retrospectively registered).

## Administrative information

Note: The numbers in curly brackets in this protocol refer to SPIRIT checklist item numbers. The order of the items has been modified to group similar items (see).

**Table:** 

Title {1}	A randomized trial of low-dose thrombolysis, ultrasound-assisted thrombolysis or heparin for intermediate-high risk pulmonary embolism—the STRATIFY trial: design and statistical analysis plan.
Trial registration {2a and 2b}.	Ethics Committee approval: H-18013257 Danish Medicines Agency: 2017–005075-91 Data Protection Agency: VD-2019–52
Protocol version {3}	Version 1.7 (24OCT2023)
Funding {4}	The Danish Heart foundation: 225,000 dkk. Brødrene Hartmanns foundation: 100,000 dkk. Jascha foundation: 220,000 dkk. Grosserer L.F. Foghts foundation: 100,000 dkk. Karen Elise Jensen foundation: 465,000 dkk. Gangsted foundation: 620,000 dkk. Skibsreder Per Henriksens foundation: 250,000 dkk. Rigshospitalets Research foundation: 610,000 dkk.
Author details {5a}	Department of Cardiology, The Heart Centre, Copenhagen University Hospital Rigshospitalet, Copenhagen, Denmark
Name and contact information for the trial sponsor {5b}	Jesper Kjaergaard, consultant, MD, PhD, DMSc, Department of Cardiology, The Heart Centre, Copenhagen University Hospital Rigshospitalet, Copenhagen, Denmark Jesper.kjaergaard.05@regionh.dk
Role of sponsor {5c}	This is a sponsor-investigator-initiated study with no funding or involvement from pharmaceutical companies, and the sponsor-investigator maintains authority over all aspects of the trial including design, management, interpretation of results and publication.

## Introduction

### Background and rationale {6a}

Thrombolysis for pulmonary embolism (PE) is a well-established, guideline-recommended therapy for high-risk PE, defined by the presence of shock. Intermediate-risk PE is stratified into intermediate high-risk or intermediate-low risk, where high-risk patients have demonstrated a significant risk of clinical deterioration and death in several cohorts [1]. Conservative treatment strategies do not seem to prevent deterioration in these patients, and thus alternative and more aggressive treatment strategies have been investigated.

Systemic high-dose thrombolysis for intermediate-high risk PE remains a matter of discussion, even though this treatment has been studied in several randomized clinical trials. The 1997 trial by Dr Konstantinides et al. and the “Fibrinolysis for patients with intermediate-risk pulmonary embolism (PEITHO)” trial from 2014 both showed a significant reduction in risk of clinical deterioration. However, in both trials, overall mortality was similar in the group treated with thrombolysis and placebo because of risk of bleeding, including risk of fatal bleeding, was substantially increased with thrombolysis [2, 3].

Since balancing of effect and complications was not successful with high-dose thrombolysis for this group, different treatment approaches including reduced dose of thrombolysis have been suggested in intermediate-high risk PE; however, consistent results have not yet been published.

#### Low-dose thrombolysis

Low-dose thrombolysis has been introduced as the concept of administering reduced concentration of thrombolysis over a similar or longer period, thus reducing the concentration of thrombolytic in the patient bloodstream at any given time point.

Several smaller studies have tested low-dose thrombolysis in PE patients. A study of 90 patients with acute PE compared 0.6 mg/kg Alteplase (rtPA) over 15 min with high dose rtPA of 100 mg over 2 h, with no differences in clot burden at repeat angiographies [4]. A later study by Wang et al. of 118 patients allocated to 50 mg/kg or 100 mg/kg rtPA over 2 h, found similar efficacy but also suggested that reduced dose rtPA seemed to have a better safety profile [5].

Only a few prospective randomized trials have been performed: In 1990 Levine et al. performed a randomized study of 0.6 mg/kg rtPA over 2 h compared with anticoagulation alone [6]. This study found a 37% improvement in lung perfusion compared to 18% in the anticoagulation group [6].

Recently a trial compared a “safe dose” of rtPA (50 mg over 2 h) in addition to anticoagulation alone [7]. The trial showed a significant reduction in incidence of pulmonary hypertension and recurrent PE at follow-up, but no differences in total mortality. The effect on clot burden was not assessed. There were no bleeding complications in either group [7].

Thus, reduced or low-dose thrombolysis has been tested and seems to be safe, but very low doses of rtPA as introduced with the recent ultrasound-assisted thrombolysis trials have not been tested in PE patients. These trials have applied a 20-mg dose over a period of 15 h [8, 9], and another trial presented at a conference in May 2017 has applied even lower doses in combination with ultrasound-assisted thrombolysis.

In conclusion, low-dose thrombolysis (approximately 50 mg of rtPA over 2 h) seems safer than high-dose thrombolysis. The efficacy may be smaller but seems to be better than anticoagulation alone.

#### Ultrasound-assisted thrombolysis

Catheter-based techniques for clot removal or fragmentation have been introduced in recent years and several specialized catheters have been developed for short-term application. Case reports and series have also shown that catheter-based fragmentation without thrombolysis may be efficacious [10, 11].

The addition of ultrasound-emitting crystals to catheter-based intervention (USAT) seems to increase the efficacy of thrombolysis. This technique has been applied in one randomized trial of 58 patients with acute PE finding a more rapid normalization of the ratio between right and left ventricle (RV/LV ratio) and reduction in pulmonary artery (PA) pressures [9]. Later, several case series of USAT application in patients with high-risk and intermediate-high risk PE have been published, suggesting a good efficacy and an acceptable safety profile with a bleeding risk of approximately 10% [8, 12].

In conclusion, catheter-based techniques have been applied in managing PE for decades with good efficacy. The current knowledge from comparative randomized trials is sparse, and the additional efficacy of catheter-based techniques is largely unknown. With recent ultrasound-based techniques combined with low-dose thrombolysis, a new approach for managing patients at risk of clinical deterioration and death has emerged. Since the risk of complications with full-dose thrombolysis has proven to balance the efficacy, USAT may be a reasonable alternative. However, the net efficacy of applying this invasive technique over low-dose thrombolysis alone is unknown and should be assessed in trials to further optimize the treatment of intermediate-high risk patients.

### Objectives {7}

This clinical randomized trial investigates the clot burden reduction potential of low-dose thrombolysis (20 mg rtPA over 6 h) compared to heparins in patients with intermediate-high risk acute PE. As a co-primary endpoint, the trial assesses the incremental efficacy of USAT administration of low-dose rtPA compared to systemic administration of 20 mg of rtPA.

Thus, the two main hypotheses being tested are:

1. Administration of 20 mg of rtPA is associated with a reduction in clot burden compared with heparins in patients with intermediate-high risk PE
2. Administration of 20 mg of rtPA as part of a USAT strategy is associated with an increased reduction in clot burden compared with systemic administration of 20 mg rtPA in patients with intermediate-high risk PE

### Trial design {8}

Multicenter, randomized clinical design allocating intermediate-high risk PE patients in a 1:1:1 ratio to one of three different treatment strategies: (1) standard treatment with heparin, (2) heparin + 20 mg rtPA over 6 h intravenous (IV), or (3) heparin + 20 mg rtPA over 6 h via USAT. The trial is a superiority trial, aiming to show a reduction in pulmonary vascular obstruction as the trial’s main endpoint. The degree of pulmonary vascular obstruction will be assessed by the application of a refined Miller score (RMS), which has been developed and validated for use in patients with intermediate and high-risk PE [14]. The original Miller index was developed for pulmonary angiography in the 1970s [13] and was later redesigned to be applied for computed tomography pulmonary angiogram (CTPA), which has become the modern standard for diagnosing PE [15]. The RV/LV ratio will be measured according to the methods described by Ouriel et al. [14] which is a slightly modified version of the methods described by Quiroz et al. [16].

## Methods: participants, interventions, and outcomes

### Study setting {9}

Trial patients are included at The Department of Cardiology, Copenhagen University Hospital, Rigshospitalet, Copenhagen, Denmark, The Department of Cardiology, Bispebjerg Hospital, Denmark, The Department of Cardiology, Gentofte Hospital, Denmark, and the Department of Cardiology, Herlev Hospital, Denmark. All USAT procedures are performed at Rigshospitalet, Copenhagen, Denmark.

### Eligibility criteria {10}

The definition of intermediate-high risk PE is based on the European Society of Cardiology (ESC) guideline classification from 2019 (1) as an identification of PE in the pulmonary main trunk or segmental pulmonary arteries on CTPA, absence of shock but signs of RV dysfunction, and elevated cardiac biomarkers.

RV dysfunction is defined as:

- RV/LV ratio of > 1 on CTPA or echocardiography (apical 4 chamber view in-diastole) OR.
- RV systolic function by visual assessment or tricuspid annular plane systolic excursion (TAPSE) < 18 mm OR.
- Tricuspid regurgitation (TR) gradient > 40 mmHg.

Elevated cardiac biomarker is defined as:

- Increase in high sensitive cardiac troponins I or T (TnI/TnT) above normal OR.
- Increase creatine kinase MB (CKMB) above normal OR.
- Increase in N-terminal Pro-B-type natriuretic peptide (NT-pro-BNP) above normal.

The absence of shock at the time of screening is defined as:

- Systolic blood pressure > 100 mmHg.

#### Inclusion criteria

1. Age ≥ 18 years.
2. Informed consent for trial participation.
3. Intermediate high-risk PE according to ESC criteria.
4. Thrombus visible in main, lobar, or segmental pulmonary arteries on CTPA.
5. Fourteen days of symptom duration or less.

#### Exclusion criteria

1. Altered mental state (Glasgow Coma Scale < 14).
2. No qualifying CTPA performed (> 24 h since CTPA).
3. Females of childbearing potential, unless negative human chorionic gonadotropin test is present.
4. Thrombolysis for PE within 14 days of randomization.
5. Thrombus passing through patent foramen oval (risk of paradoxical embolism) seen on echocardiography.
6. Ongoing oral anticoagulation therapy (heparins, aspirin, antiplatelet therapy, and NOAC allowed).
7. Comorbidity making 6 months survival unlikely.
8. Absolute contraindications for thrombolysis.

- Haemorrhagic stroke or stroke of unknown origin at any time.
- Ischemic stroke in the preceding 6 months.
- Central nervous system damage or neoplasms.
- Recent major trauma/surgery/head injury in the preceding 3 weeks.
- Gastrointestinal bleeding within the last month.
- Known elevated bleeding risk.

Relative contraindications for thrombolysis do not preclude randomization. Relative contraindications include a transient ischemic attack in the preceding 6 months, oral anticoagulant therapy, pregnancy, or within 1-week postpartum, non-compressible puncture site, traumatic resuscitation, refractory hypertension (systolic blood pressure > 180 mm Hg), advanced liver disease, infective endocarditis, active peptic ulcer.

### Who will take informed consent? {26a}

Informed consent is obtained by the attending cardiologists at the recruiting centers, either in the emergency room or at the ward.

### Additional consent provisions for collection and use of participant data and biological specimens {26b}

The consent covers data storage and access to registry and administrative health care data; performance of two CTPAs at 48–96 h and after 3 months; a follow-up visit after 3 months and questionnaires; a collection of 10 ml of blood for storage in biobank.

## Interventions

### Explanation for the choice of comparators {6b}

All three interventions tested in the trial are in line with current treatment guidelines; however, the optimal treatment strategy for patients fulfilling the trial inclusion criteria has yet to be identified.

### Intervention description {11a}

All patients will be treated according to current national guidelines at the discretion of the treating physician. Long-term anticoagulation will be planned by the treating physician according to patient preferences. As a minimum, oxygen saturation, blood pressure, heart rate, and telemetry should be measured every hour for the first 24 h.

At the time of the diagnosis of intermediate-high risk PE, it is expected that most patients will be treated with unfractionated heparin (UFH) or Low molecular weight heparin (LMWH) according to site protocol. As per national guidelines, this adheres to the “wait-and-see” approach to this patient population in whom a risk of deterioration is present, which may require immediate full-dose thrombolysis or surgical embolectomy. To allow for such an up-scaling in treatment, anticoagulation with heparins is continued for at 24–48 h as per the discretion of the treating physician.

Inclusion in the trial will thus add treatment with low thrombolysis with or without USAT in two-third of the patient population.

#### Low-dose thrombolysis for IV infusion

For systemic thrombolysis, 20 mg of rtPA is dissolved in 20 ml of sterile water and further diluted into a total volume of 250 ml isotonic saline. The rtPA solution is infused in each catheter over 6 h (infusion rate approx. 41 ml/h) in a separate IV line.

#### Low-dose thrombolysis as part of USAT

Patients allocated to low-dose thrombolysis via USAT are transferred to Rigshospitalet as soon as possible, in order to initiate treatment with USAT within 12 h post-randomization. Two USAT catheters are placed in the lower pulmonary artery under fluoroscopic guidance via access from the femoral vein. Vein puncture is performed under ultrasound guidance, and 2 6 Fr. sheaths are placed using the Seldinger technique.

Immediately after placing the catheter, the ultrasound-emitting crystal catheter is placed in the USAT catheter, and an infusion of 10 mg rtPA (20 mg vial, separated into 2 × 250 ml isotonic saline solution) is administered over 6 h (infusion rate 41 ml/h). Further 30 ml/h of isotonic saline infusion as a coolant is infused via the catheters.

As most patients are expected to have bilateral clots, two USAT catheters are placed. If only unilateral PE is found, the 20 mg of rtPA is infused in the catheter instead of the 10 mg for bilateral catheters.

After 6 h the infusion of rtPA is terminated, and the USAT catheters are removed, leaving the sheaths in place. After an additional 2 h, the sheaths are removed under manual compression of the puncture site for 10 min. One hour after removal of the sheaths the patient is confined to bed with a maximum of 30 degree elevation of head rest. Analgesics and sedatives (benzodiazepines) may be ordered for patients’ comfort.

Patients are transferred to Rigshospitalet after initiation of heparin. If LMWH administration is less than 6 h away, no bolus UFH dose is given. Otherwise, a bolus dose of 60 IE/kg (max. 4000 IE) is given, followed by an infusion of 12 IE/kg/h (max. 1000 IE/h as initial infusion rate). The UFH is administered in a separate IV line. Infusion rates are adjusted according to Activated partial thromboplastin clotting time (APTT), targeting 1.5–2.5 times longer APTT than the baseline value. APTT is measured every 4 h for the first 8 h, and then longer time intervals can be ordered. Activated Clot Time (ACT) of 160–180 s can be used as an alternative.

The patients transferred to Rigshospitalet were admitted here until the 48–96-h CTPA was performed and then transferred back to the local hospitals. All other participating sites were level 2 with specialized cardiologists attending.

### Criteria for discontinuing or modifying allocated interventions {11b}

If local reaction or bleeding occurs when treated with rtPA IV or via USAT, the infusion is paused, and the attending physician will decide whether to terminate the infusion or not. The T_1/2_ of rtPA is 5 min.

If a patient clinically deteriorates despite relevant treatment, whether it is heparin or heparin and low-dose thrombolysis IV/USAT, upscaling treatment to full-dose thrombolysis (rtPA 100 mg IV over 2 h) will be initiated in accordance with current guidelines.

### Strategies to improve adherence to interventions {11c}

All physicians and nursing staff involved in the care of the study patients are trained on a regular basis in the allocated treatment strategies and obtainment of relevant endpoints. The steering group is available for advice if needed by the treating physician.

### Relevant concomitant care permitted or prohibited during the trial {11d}

The study intervention ends after completion of follow-up CTPA conducted 48–96 h post-randomization. Concomitant care adheres to established clinical standard protocols including transition to oral anticoagulant treatment.

### Provisions for post-trial care {30}

Upon hospital discharge, patients are invited for a 3-month follow-up at Rigshospitalet. The follow-up includes CTPA, transthoracic echocardiography, blood sampling, 6MWT, and questionnaires.

### Outcomes {12}

#### Co-primary endpoint

- Reduction in refined Miller score (RMS) at 48–96 h post-randomization (score of thrombus involvement and segmental flow) [13, 14] comparing thrombolysis groups (combining groups with and without USAT) to UFH/LMWH group, *p* < 0.01 (two-sided), full study population, *n* = 140 vs. *n* = 70.
- Reduction in RMS at 48–96 h post-randomization comparing thrombolysis administered via USAT or IV, *p* < 0.04 (two-sided), population treated by low-dose thrombolysis, *n* = 70 vs *n* = 70.

#### Secondary endpoints

- Bleeding complications (major and minor bleeding complications according to the thrombolysis in myocardial infarction classification)
- Duration of index admission, including hospital-based rehabilitation
- Dyspnoea index (visual analog scale) after 48–96 h and after 3 months
- FiO2, blood pressure, and respiratory rate, heart rate at the time of follow-up CTPA
- Mortality in the three groups (log-rank), and hazard ratio in multivariable analysis using the UFH/LMWH as a reference.
- Incidence of TR gradient > 40 mmHg at 3 months follow-up echocardiography
- 6MWT at 3 months follow up comparing the three groups.
- Quality of life at 3 months follow-up comparing the three groups (PEmb-Qol and 5Q-5D-5L)
- We were unable to systematically collect data for the following pre-planned endpoints: reduction in D-dimer from baseline to 48–96 h post-randomization, Relative reduction in TnI/T from baseline to 48–96 h post-intervention, and reduction in NT-pro-BNP at 48–96 h and 3 months. These secondary endpoints will not be reported in the primary publication.

We were unable to systematically collect data for the following pre-planned endpoints: reduction in D-dimer from baseline to 48–96 h post-randomization, Relative reduction in TnI/T from baseline to 48–96 h post-intervention, and reduction in NT-pro-BNP at 48–96 h and 3 months. These secondary endpoints will not be reported in the primary publication.

### Participant timeline {13}

**Fig. 1:**
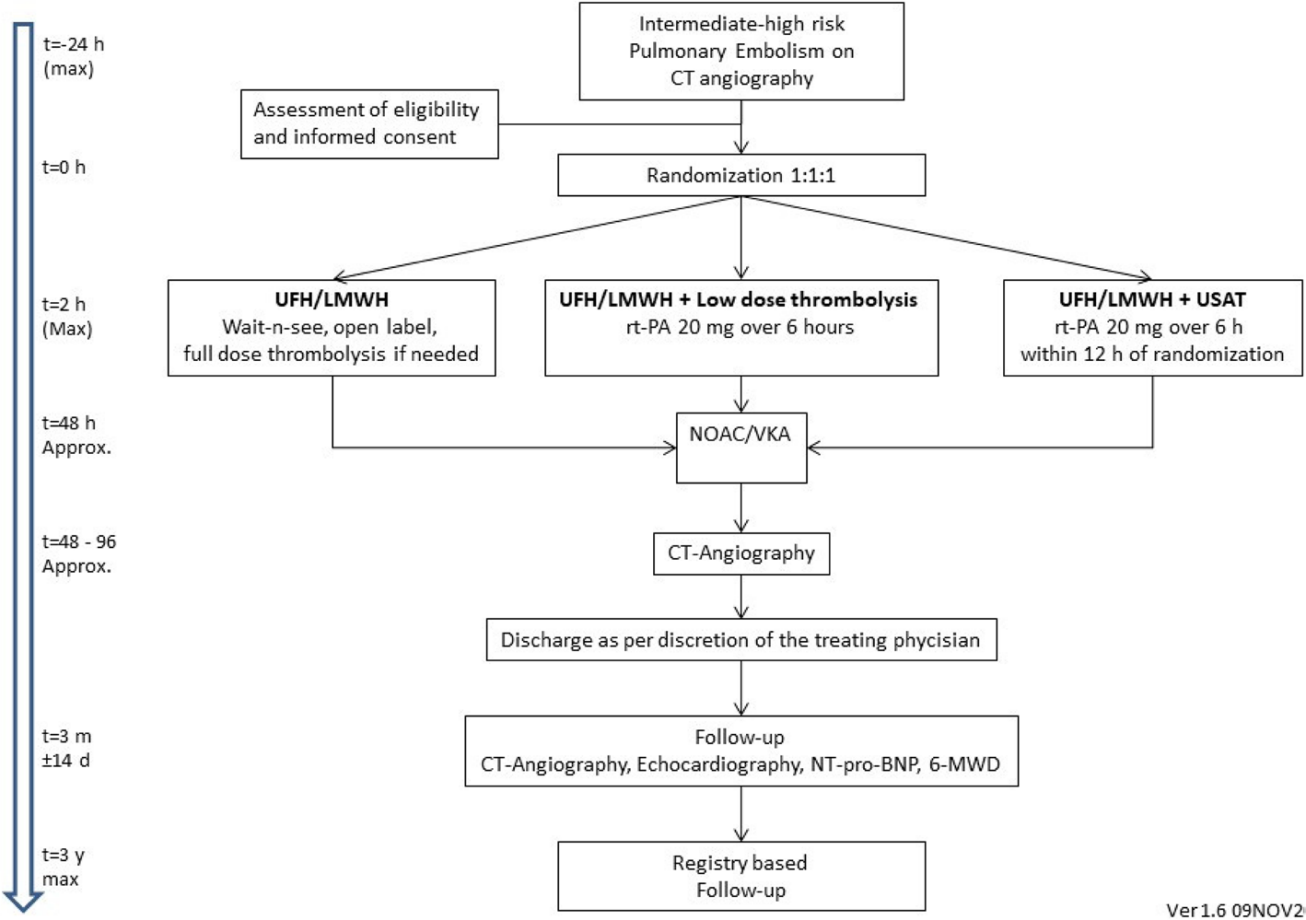
Flowchart of patient enrolment and participation. CT, computed tomography; UFH, unfractionated heparin; LMWH, low molecular weight heparin; USAT, ultrasound-assisted thrombolysis; NOAC, novel oral anticoagulation; VKA, vitamin K antagonist; h, hour; m, month; y, year

The flowchart is shown in Fig. 1.

### Sample size {14}

The power of the trial is calculated as two co-primary endpoints, sharing a combined α-level of 0.05.

A mean RMS of 18 ± 7 is expected at baseline [14]. A variance component model (proc mixed, SAS enterprise ver. 8,4, Cary, NC) was applied.

The treatment effect is expected to be a reduction in RMS of 2 points (11%) in the UFH/LMWH group after 48–96 h (extrapolation from [13]), whereas the reduction in RMS in both thrombolysis groups is expected to be 6 points (33%) [13]. The net effect of thrombolysis by USAT is expected to be similarly efficacious.

The first co-primary endpoint evaluates the effect of the intervention of thrombolysis comparing RMS in the UFH/LMWH group to RMS in the combined groups of low-dose thrombolysis with or without USAT (1:2).

Planning inclusion of 165 (3*55) patients will give a power of 0.8 whereas 210 (3*70) patients will give a power of 0.90, with an α of 0.01 for the primary endpoint.

The second co-primary endpoint evaluates the effect of low-dose thrombolysis administered by USAT or IV will yield a power of 0.9 by the inclusion of 140 patients (1:1) with an α-level of 0.04, assuming a 22% reduction or greater reduction in RMS in the USAT group compared the IV group.

The trial is planning to include 210 patients, with 70 patients assigned to UFH/LMWH, 70 to low-dose thrombolysis without USAT, and 70 assigned to low-dose thrombolysis with USAT initiated within a mean of 12 h from randomization.

### Recruitment {15}

Each inclusion site is routinely visited by a member of the steering group who provides updates and trains new staff members on patient screening procedures and study protocol, thereby ensuring consistent patient enrollment.

## Assignment of interventions: allocation

### Sequence generation {16a}

Patients are allocated to treatments in a 1:1:1 manner through computer-generated randomization.

### Concealment mechanism {16b}

Inclusion and randomization are performed via a dedicated website, and the allocation is kept concealed until the time of randomization.

### Implementation {16c}

The attending physicians at inclusion sites will have access to the trial inclusion website and will thus clarify inclusion and exclusion criteria and include patients in the trial. A consent from the assigned will automatically be requested from the webpage.

## Assignment of interventions: blinding

### Who will be blinded {17a}

The assessment of RMS will be made by trained radiologists who will be kept blinded to the timing of CTPA (whether diagnostic or follow-up) and the allocated treatment. Since the follow-up CTPA is performed after removal of catheters for USAT and IV lines for systemic low-dose thrombolysis the risk of unblinding is limited.

### Procedure for unblinding if needed {17b}

A person will be available for unblinding if needed. The allocation groups are open label; therefore, we do not expect a need for unblinding.

## Data collection and management

### Plans for assessment and collection of outcomes {18a}

All patients diagnosed with an intermediate-high risk PE at the participating site will be screened for eligibility for inclusion in the trial. A specific web-based database for this will be constructed and made available for the participating sites.

The assessment of RMS will be made by trained radiologists who will be kept blinded to the type of CTPA (whether diagnostic or follow-up) and the allocated treatment. Since the follow-up CTPA is performed after removal of catheters for USAT and IV lines for systemic low-dose thrombolysis the risk of unblinding is limited.

The assessment will be entered into a database with access restricted to the assessor. Patients will be identified by the ID number (CPR) and the CTPA by the time and date of the CTPA.

Data apart from the screening form and the clot burden assessment will be collected by study personnel at Rigshospitalet by directly entering into a trial database, using patients’ medical charts (SundhedsPlatformen) as data source. The trial database will be constructed using REDCap software, hosted by the Capital Region. This database complies with Danish data safety legislation.

**Fig. 2:**
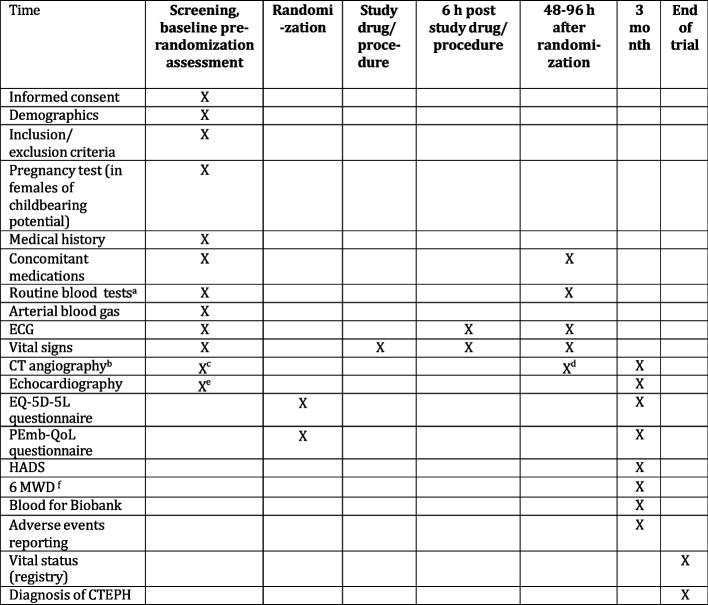
SPIRIT schedule of enrolment, interventions, and assessments. ^a^ Hemoglobin, thrombocyte count, leucocytes, count, renal function, troponin I/T, CKMB, CK, D-dimer, INR, APTT, liver function test, NT-pro-BNP. Blood test up to 12 h prior to randomization are considered valid. ^b^ CT angiography according to local protocol. eGFR < 30 preclude CT angiography. ^c^ CT angiography for diagnosis is used if < 24 h from the screening time point. ^d^ CT angiography ordered 48–96 h post-randomization as convenient. ^e^ Standard TTE, including TR gradient. ^f^ 6-min walk distance (m), CTEPH, chronic thromboembolic pulmonary hypertension; HADS, Hospital Anxiety and Depression Scale

Informed consent forms will be scanned and stored on the patients’ chart (SundhedsPlatformen) and the Alteplase batch number will be documented by photo in the SundhedsPlatformen media section (Fig. 2).

### Plans to promote participant retention and complete follow-up {18b}

A dedicated member of the steering group is responsible for overseeing participant retention and ensuring the completion of follow-up procedures for patients across all trial sites. Prior to each scheduled follow-up visit, patients receive a telephone call to confirm the date and location. While no financial support is provided for visitation, transportation expenses may be reimbursed upon request. Patients who deviate from the study protocol will be retained in the study database unless they choose to withdraw their informed consent.

### Data management {19}

Study data are stored in a REDCap database managed by the Regional IT department, with entry conducted by personnel at Rigshospitalet. The primary investigator performs random sampling to ensure quality control, while a GCP monitor reviews and validates consent forms, eligibility criteria, and study endpoints.

### Confidentiality {27}

Confidentiality of patients’ information is ensured using a REDCap database, and furthermore, all information will be accessed and handled by trained medical staff with valid authorization.

### Plans for collection, laboratory evaluation, and storage of biological specimens for genetic or molecular analysis in this trial/future use {33}

A biobank with 10 ml of blood per patient will be established. The purpose of the biobank is to be able to examine other hypotheses after a renewed application for permission from the Science Ethics Committee and renewed consent from the patient. The biobank is included in the informed consent. All samples from the biobank are handled anonymously. The biobank’s material is destroyed 20 years post end of the trial, i.e., December 1, 2044.

## Statistical methods

### Statistical methods for primary and secondary outcomes {20a}

The baseline characteristics of the patients will be compared in the three treatment allocation strata. Two tests for each characteristic will be performed: one comparing group 1 to group 2/3 (primary outcome) and one comparing groups 2 and 3 (co-primary outcome).

The RMS will be at baseline and at the follow-up CTPA in groups 1 and 2/3 as the first co-primary endpoint. Site is defined as the site of randomization.

In the statistical model, the relative change adjusted for baseline value and the statistical significance for a difference between groups 1 and 2/3 will be tested in a variance component model adjusted for site.

The model statement will be RMS_48–96h_ = RMS_baseline_ + group 2/3 vs. 1 + site. Patients dying before the follow-up CTPA will be assigned the highest RMS score in the stratum.

For the second co-primary endpoint analysis the mean RMS for group 2 vs. 3 will be described, as the mean change from baseline in group 2 vs. 3. Differences will be tested in a variance component model adjusted for site with the following parameter: RMS_48–96h_ = RMS_baseline_ + group 2 vs. 3 + site.

Patients dying before the follow-up CTPA will be assigned the highest RMS score in the stratum.

The remaining secondary endpoints will be analyses comparing group 2/3 to group 1 as well as comparing group 2 vs. group 3.

Secondary outcomes will be assessed using the chi-squared test for dichotomous variables and the ANOVA test for continuous variables.

### Interim analyses {21b}

The Data and Safety monitoring board (DSMB) is chaired by ass. Professor Jens Jakob Thune MD PhD has been overseeing the trial adverse events. No formal interim analyses were planned. The DSMB has full authority to plan and request safety analyses. The DSMB has not had any concerns regarding the trial conduct and recommends the trial be completed as planned.

### Methods for additional analyses (e.g., subgroup analyses) {20b}

The following are predefined design variables that will examined in subgroup analyses:

1. Sex
2. Age above median
3. Known renal failure (GFR < 30 ml/min or current renal replacement therapy)
4. Known chronic obstructive pulmonary disease
5. “Saddle” embolus at CT angiography
6. Syncope before randomization
7. Cardiopulmonary resuscitation performed before randomization

### Methods in analysis to handle protocol non-adherence and any statistical methods to handle missing data {20c}

Trial sites will be asked to complete all CRFs and other forms if missing data is found in the electronic database. Missing data will be reported in the publication. Analyses will be performed according to the modified intention to treat principle.

### Plans to give access to the full protocol, participant-level data and statistical code {31c}

Access to the protocol can be granted upon request. If access to participant-level data is required, permission from the data regulation authorities must be obtained.

## Oversight and monitoring

### Composition of the coordinating center and trial steering committee {5d}

The trial steering committee consists of Jesper Kjærgaard (JK), Jørn Carlsen (JC), Lene Holmvang (LH), Jens Flensted Lassen (JFL), Rikke Sørensen (RS), Peter Sommer Ulriksen (PSU), Ole P Kristiansen (OPK), Kristian Eskesen (KE), Søren Fanø (SF), Emilie Sonne-Holm (ES-H), and Lia E. Bang (LB). This group represents the four sites participating in the trial. Rigshospitalet is the coordinating center.

The Good Clinical Practice (GCP) unit at the research department of the Cardiology Research Unit oversees that the study is performed according to the principles of good clinical practice. The GCP unit reports to the study sponsor.

Principle Investigator (PI) (JK): sponsor, coordination of protocol development, funding, ethical approval, information, recruitment of trial sites, daily management, and authorize invoices.

Steering group: Protocol development, funding, information, recruitment of trial sites, sub-study coordination.

Site investigators (OPK, SF, KE, LB): responsible for all trial-related procedures at their site, availability of the study drug, training on monitoring of study drug preparation quality and protocol adherence, education of staff in trial-related procedures, recruitment, and follow-up of patients. Clinical staff at the trial sites will perform treatment of trial patients.

Operational management group (JK, ES-H, JC, LB): daily management, approve new trial sites, discontinue trial sites, take responsibility as PI for limited time periods.

Independent Data Safety Monitoring Committee (JJT): evaluates SAEs and SUSARs and perform interim analyses. Will provide recommendations about stopping, halting, or continuing the trial to the trial steering group.

### Composition of the data monitoring committee, its role and reporting structure {21a}

The DSMB board consists of Ass. Professor Jens Jakob Thune, Department of Cardiology, Bispebjerg Hospital. The DSMB defines the need for interim analyses independently and reports back to the Steering Committee if needed. The DSMB chair is free and includes other members in the work of overseeing the trial.

### Adverse event reporting and harms {22}

Reporting of adverse events will be in accordance with the principles of Good Clinical Practice. Abnormal laboratory values should not be reported as adverse events; however, any clinical consequences of such abnormal values should be reported as adverse events.

### Frequency and plans for auditing trial conduct {23}

The trial will be monitored by the Good Clinical Practice unit at Frederiksberg Hospital.

### Plans for communicating important protocol amendments to relevant parties (e.g., trial participants, ethical committees) {25}

All protocol revisions have been communicated to the ethics committee as required. No updates to the current protocol are planned, besides updating the trial status.

### Dissemination plans {31a}

The main trial paper manuscript will be drafted according to the statistical analysis plan by the Rigshospitalet Steering group members. Authors of the main trial manuscript are PI (first author), top including site steering group members, top including site runner-up steering group members, remaining steering group members, and Steering Group senior author. Data Safety Monitoring Board members will be offered co-authorship. The target journal will be a high-ranking international medical journal.

The main trial results will be published regardless of being positive, neutral, or negative. The main trial will also be submitted for presentation at national and international conferences.

## Discussion

As described, the optimal treatment strategy for patients fulfilling the trial inclusion criteria has yet to be identified. Full-dose thrombolysis is efficacious but is associated with a risk of bleeding complications, largely outweighing the benefits of this approach.

Low-dose thrombolysis is promising regarding efficacy and lower risk of bleeding complications, but the amount of data from case series and trials are still very limited. Furthermore, the optimal dose within the range of doses defining low-dose thrombolysis has not yet been identified. As mentioned, the existing trials have used approximately half-dose regimens, but with the introduction of catheter-based techniques, including USAT, the dose is even lower. In many studies, approximately 1/5 of the standard full dose regimen and infusion was used. The risk of major bleeding, including intracranial bleeding, is low in general (< 5%) and expected to be even lower in the present study due to the reduced doses used.

The catheter-based techniques are invasive in nature, and although an increasing amount of data suggests their efficacy, the bleeding rates are still not clinically irrelevant. Further, as the procedure involves mechanical fragmentation by placing the catheter as well as ultrasound-emitting crystals supposedly increasing efficacy of the low-dose thrombolysis, knowledge of which part is the more efficient is needed. In fact, the current trial may show that the USAT procedure is no more efficacious than low-dose systemic thrombolysis alone. The USAT technique has been used for the past 2 years in patients with intermediate-high risk PE, and the rate of complications in our experience is equal to that of the published case series: approximately 10% have minor or major bleeding complications and the reduction in clot burden and clinical effect of treatment is present.

In conclusion, a clinical equipoise exists between the three treatment strategies in the current trial. Bleeding risk associated with low-dose thrombolysis or USAT procedure are small and acceptable compared to the risk of clinical deterioration, and all treatment strategies studied are within current treatment guidelines, acknowledging that the dose of the rtPA in the systemic thrombolysis group has been tested previously. However, the dose used in this trial is even lower, and therefore the risk of complications is expected to be similar to the published literature or lower.

Additional investigation at the 3-month follow-up visit will have minimal ethical implications. Questionnaires are standard questionnaires developed for this purpose; 6MWT is symptom-limited and considered to be with no hazard. Slight discomfort from the exercise is expected but temporary and self-limiting in nature as with other exertional efforts. The blood draw for the biobank is performed in conjunction with the clinical routine blood draw and 10 ml additional blood volume draw poses no risk or additional hazard or discomfort to the patient. Two extra CTPAs are performed, one during the index admission and one in conjunction with the 3 months follow-up visit. The two CTPAs will be associated with a total extra radiation dose of no more than 20 mSv, corresponding to 6 years in background radiation, and may be associated with a minimal increase in lifetime cancer risk. The research biobank will be stored until 31. March 2038 before being transferred to a biobank.

The trial will thus provide results that will help optimize treatment strategies for future intermediate-high risk PE patients**.**

## Trial status

Protocol Version 1.7 (24OCT2023).

The first patient was enrolled on the 6th of June 2019. The last patient is expected to be included before August 2024. Three months follow-up will then be completed before December 2024. Statistical analysis will be performed hereafter.

## Data Availability

Based on approval from authorities, data required to support the protocol can be supplied on request.

## Abbreviations

PE: Pulmonary embolism
rtPA: Alteplase
USAT: Ultrasound-assisted catheter-based thrombolysis
RV: Right ventricle
LV: Left ventricle
PA: Pulmonary artery
ESC: European Society of Cardiology
CTPA: Computed tomographic pulmonary angiography
IV: Intravenous
TAPSE: Tricuspid annular plane systolic excursion
TR: Tricuspid regurgitation
TnI: Troponin I
TnT: Troponin T
CKMB: Creatinin kinase MB
NT-pro-BNP: N-terminal pro-B-type natriuretic enzyme
UFH: Unfractionated heparin
LMWH: Low molecular weight heparin
aPTT: Activated partial thromboplastin clotting time
6MWT: 6-Min walk test
RMS: Refined Miller score
